# Serological and molecular surveillance of West Nile virus in domesticated mammals of peninsular Malaysia

**DOI:** 10.3389/fvets.2023.1126199

**Published:** 2023-06-29

**Authors:** Mohammed Nma Mohammed, Abd Rahaman Yasmin, Siti Zubaidah Ramanoon, Mohd Adzahan Noraniza, Peck Toung Ooi, Mohd Yuseri Ain-Najwa, Jafar Ali Natasha, Saulol Hamid Nur-Fazila, Siti Suri Arshad, Hussni Omar Mohammed

**Affiliations:** ^1^Department of Veterinary Laboratory Diagnosis, Faculty of Veterinary Medicine, Universiti Putra Malaysia, Serdang, Malaysia; ^2^Department of Animal Production, School of Agriculture and Agricultural Technology, Federal University of Technology, Minna, Nigeria; ^3^Laboratory of Vaccines and Biomolecules, Institute of Bioscience, Universiti Putra Malaysia, Serdang, Malaysia; ^4^Department of Farm and Exotic Animal Medicine and Surgery, Faculty of Veterinary Medicine, Universiti Putra Malaysia, Serdang, Malaysia; ^5^Department of Veterinary Clinical Studies, Faculty of Veterinary Medicine, Universiti Putra Malaysia, Serdang, Malaysia; ^6^Department of Veterinary Pathology and Microbiology, Faculty of Veterinary Medicine, Universiti Putra Malaysia, Serdang, Malaysia; ^7^Department of Population Medicine and Diagnostic Sciences, College of Veterinary Medicine, Cornell University, Ithaca, NY, United States

**Keywords:** West Nile virus, cattle, goat, horse, c-ELISA, antibodies, RT-PCR, zoonosis

## Abstract

West Nile virus is a mosquito-borne neurotropic pathogen with a wide host range that constitutes a significant risk to public and animal health. There is limited information on WNV infection in domesticated mammals in Malaysia; however, current reports indicate infections in birds, macaques, bats and pigs from Malaysia. In this study, 203 serum samples from cattle, goats, and horses were tested for the presence of anti-WNV IgG using a competitive enzyme-linked immunosorbent assay (c-ELISA). Additionally, using one-step RT-PCR, nasopharyngeal swabs were analyzed for WNV RNA from all 203 animals in this study. The WNV seroprevalence was 32.53% (27/83) at 95% CI (0.2342–0.4319) in cattle, 48.27% (14/29) at 95% CI (0.3139–0.6557) in goats and 53.84% (49/91) at 95% CI (0.4366–0.6373) in horses. Cross-reactive JEV antibodies were detected in two cattle and 34 horses. None of the cattle or goats tested positive for WNV RT-PCR. Seven horses were positive for WNV RT-PCR, a molecular prevalence of 7.69% (7/91) at 95% CI (0.0353–0.1528). This is the first reported detection of WNV in domesticated mammals of Malaysia, a significant addition to the growing evidence that WNV is being transmitted from vectors to susceptible hosts in Malaysia.

## 1. Introduction

Arboviruses represent an increasing threat to global public health due to their ability to establish and spread rapidly once introduced into new areas with abundant competent vectors and susceptible hosts (1). Members of the flavivirus genus are transmitted by vectors, notably by mosquitos and ticks. The genus is comprised of several important human and animal viruses like the yellow fever virus (YFV), dengue virus (DENV), Japanese encephalitis virus (JEV), Saint Louis encephalitis virus (SLEV), and West Nile virus (WNV). Since its initial discovery in Uganda in 1937 (2), WNV has spread globally to all continents (3) and has been the cause of large outbreaks in several countries around the world (4–6). WNV was first introduced into the United States in 1999 (7–9). By 2002, the country reported 14,000 cases in horses and 4,000 in humans (1). This unprecedented spread underscore the importance of continuous surveillance and tracking the activity of WNV globally.

WNV is transmitted mainly by ornithophilic Culex mosquitos, which become viremic from feeding on infected birds that are the reservoir and amplifying host of the virus (10). The virus exists in a natural cycle between birds and mosquitos, with occasional spill-over events when infected Culex mosquitos opportunistically feed on other vertebrate species, including humans, horses, ruminants, wildlife and reptiles. WNV infection has been widely reported in horses (11–15), and there have also been some reports in goats, sheep and cattle (16–18). In these species, clinical disease leading to neurological involvement were occasionally registered. These species are dead-end hosts and are not involved in the amplification and transmission of the virus due to a low and transient viremic phase during infection. Nevertheless, infection with WNV in these species, mainly in the horse, can lead to considerable loss to animals’ owners and potential outbreaks involving other species, including humans.

Because of the tropical climate in Malaysia that supports a rich biodiversity, mosquitos, including Culex, as well as various wild bird species are abundant. Recent studies have reported the detection of WNV in birds, with seropositive rate of 18.71% (29/155) molecular prevalence of 15.23% (16/105), in macaques with WNV seroprevalence of 29.63% (24/81), in bats with WNV RNA prevalence of 12.19% (5/41) and in pigs with WNV seroprevalence of 62.5% (19–21). However, no clinical illness due to infection with WNV has been reported in Malaysia. There has been an increase in the amount of information about WNV activity; however, no information is currently available on WNV infection in horses and domesticated ruminants, including cattle and goats. This study was therefore carried out to determine the exposure to WNV in cattle, goats and horses using serological and molecular methods.

## 2. Materials and methods

### 2.1. Ethical approval

This study was carried out in accordance with approved protocols of the Institutional Animal Care and Use Committee (IACUC) of the Universiti Putra Malaysia. The IACUC approval number for this study is UPM/IACUC/AUP/−R043/2017.

### 2.2. Study area and design

**Figure 1:**
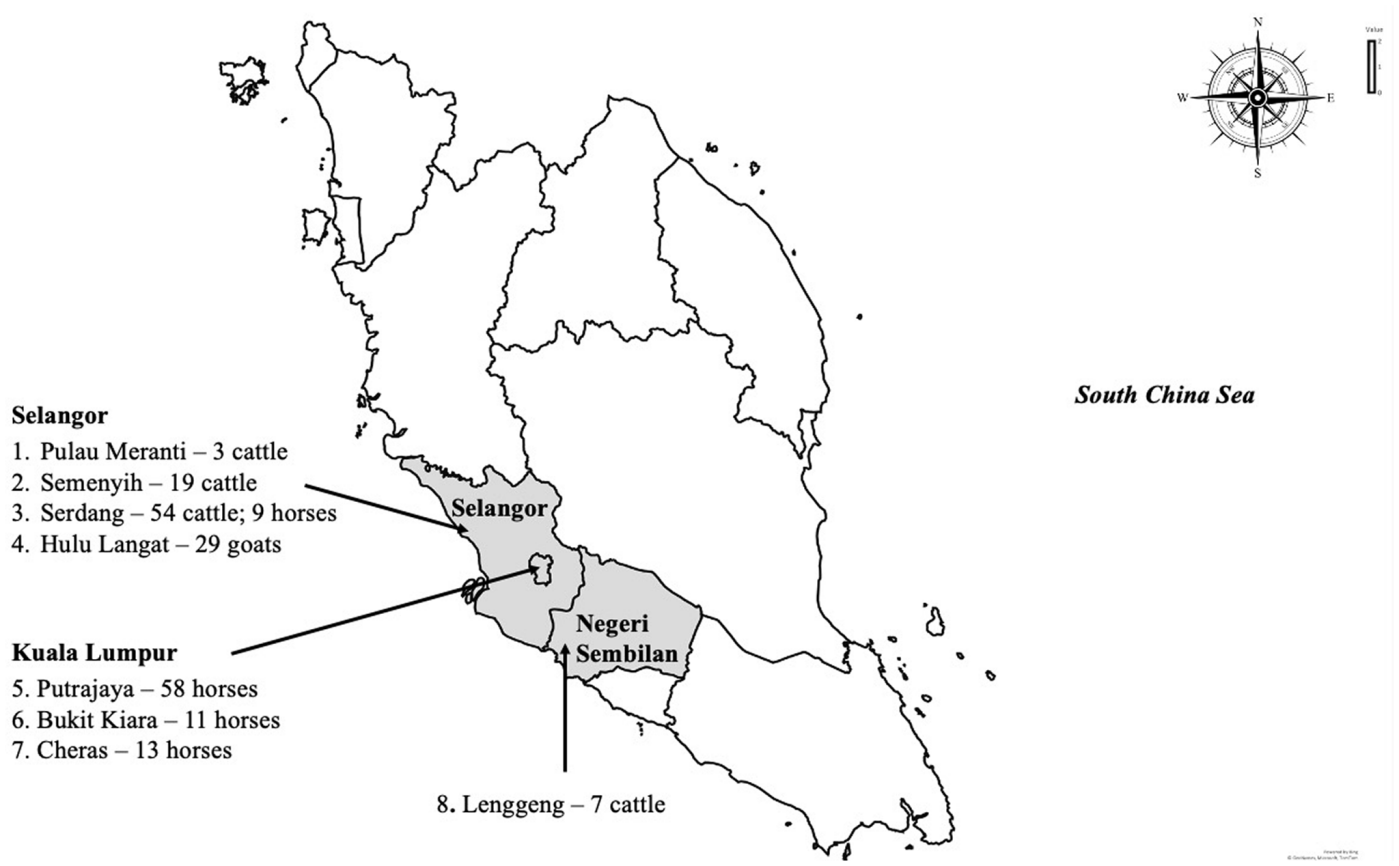
The map of Peninsular Malaysia shows the sampling locations in this study, the number and species of animals sampled.

A cross-sectional study was carried out among livestock and horses to investigate exposure to WNV in Peninsular Malaysia. Animals included in the study were those in the Faculty of Veterinary Medicine Universiti Putra Malaysia foster farms. A convenient sampling method was used in the selection of the farms and stables, while the included animals were randomly selected. In total 203 animals were sampled including cattle (*n* = 83), goats (*n* = 29) and horses (*n* = 91) for this study between 2017 and 2019. The ruminant samples (*n* = 112) were collected from five locations, namely Serdang, Semenyih, Palau Meranti, Hulu Langat and Lenggeng (Figure 1). Horses were sampled from Putrajaya, Cheras, Bukit Kiara and Serdang. The cattle and goats were kept semi-intensively on farms together with other animals, including chickens, cats, ducks and birds. Within the farm premises, pools of standing water were observed. All the horses sampled for the study were kept in well-maintained stables.

### 2.3. Sample collection

Animals from which samples were collected were randomly chosen and then were restrained. For serological assays, blood was collected through jugular (in goat, cattle and horse) or coccygeal venipuncture (in cattle). The blood was collected into a clot-activator blood collection red vacutainer tube (BD, United States). Serum was obtained by centrifuging (Eppendorf, Germany) the tubes at 2000 g for 10 min to facilitate separation. Nasopharyngeal swabs were collected from the same animals to extract whole RNA for WNV RT-PCR. A sterile cotton swab was inserted into the nasal opening of the retrained animals and gently guided in until resistance was met. It was left in place for 20 s and then gently rotated to obtain as much material as possible from the mucosal lining onto the swabs. The swab was gently withdrawn and placed in a sterile 2 mL centrifuge tube (Labcon, California) with Phosphate buffer saline (PBS) (Sigma- Aldrich, Germany) transport medium. All samples were stored at −80° C freezer (Sanyo Ultra Low, Japan) until further use.

### 2.4. WNV competitive ELISA

A commercial competitive ELISA kit (ID Screen West Nile Competition Multi-species ELISA, ID VET, France) was used for the detection of anti-WN viral envelope (E) protein antibodies (IgG) in serum of test animals. According to the manufacturer instructions, equal volumes of the test serum were added to the ELISA microplate which has its wells pre-coated with purified WNV pre-E antigen. The positive and negative controls were run in duplicates. Anti-pre-envelope horseradish peroxidase (pr-E-HRP) conjugate was added to the wells following incubation and washing of the microplates. A TMB substrate solution was added following the removal of the conjugate solution to elicit a color reaction. The optical density was measured at 450 nm (TECAN microplate reader M200, TECAN, Switzerland). The ratio of the optical density of each of the samples to that of the negative control were determined and S/N percentages were used for the interpretation of the results. Samples with an S/N percentage less than or equal to 40% were considered positive.

### 2.5. JEV double antibody sandwich ELISA

Samples positive in the WNV c-ELISA test were screened for JEV antibodies, using JEV specific double-antibody sandwich ELISA (DAS-ELISA) (Sunred Biotech, China), which detects JEV IgG in test serum. All procedures were carried out according to the manufacturer instructions. In short, test samples were diluted by adding 10 μL of the test serum to 40 μL of diluent and then added to the ELISA plate. In the control wells, 50 μL of positive control and negative control was added in duplicates. The plates were incubated at 37° C for 30 min, washed and then conjugate was added. After a 30 min incubation at 37° C the plate was washed, and chromogen A was added to each well, followed by chromogen B solution. The wells’ optical density (OD) value was read at a wavelength of 450 nm by using a TECAN microplate reader (TECAN, Switzerland). A cut-off point was determined by adding the mean of the optical densities of the negative control to 0.15 (mean ODNC +0.15). Samples with an OD equal to or greater than the cut-off value was considered positive for JEV antibodies.

### 2.6. Reverse-transcriptase polymerase chain reaction

One-step reverse-transcriptase polymerase chain reaction (RT-PCR) assay with a commercial kit (MyTaq One-Step RT-PCR, Bioline, United States) was used to detect WNV RNA from nasopharyngeal swabs obtained from the sampled animals. In summary, a primer set was designed to target a conserved region of the WNV genome as described by Ain-Najwa et al. (15). The conserved region of the WNV genome is 470 bp long between the capsid and the pre-membrane protein. The following is the sequence of the primer sets used in the amplification reaction in this study: forward primer (5′ - CCAATAC-GTTTCGTGTTGG - 3′), and reverse primer (5′ - GGAAATGACCCTGAAGACA - 3′). A synthetic cDNA of a conserved region between the capsid and pre-membrane genes of the WNV genome was used as the positive control in the amplification reaction. The total RNA was extracted from the collected nasopharyngeal swabs using triazole (TriSure, Bioline, United Kingdom). A 25 μL reaction volume (8 μL MyTaq®, 0.5 μL each of the forward and reverse primers, reverse -transcriptase enzyme and ribonuclease inhibitor, 10 μL DEPC-treated water and 5 μL template) were amplified with one-step RT-PCR using the following sequential reaction protocol: reverse transcription at 45° C for 20 min, polymerase activation at 95° C for 1 min, 10 s of denaturation at 95°C for 40 cycles, 10 s annealing at 52° C, 30 s of extension at 72° C and 5 min of final extension as the final step. The amplification reaction products were analyzed with 1.5% gel electrophoresis to determine the presence of amplicon of the expected size of 470 bp.

### 2.7. Sequencing and phylogenetic analysis

The bands visualized at the corresponding 470 bp mark were excised with sterile blades into sterile tubes, purified (Nucleospin Gel and PCR Clean-up kit, Macherey-Nagel, Germany) and sequenced (ABI PRISM 3730xl Genetic Analyzer, Applied Biosystems, United States). Phylogenetic analysis of the sequences was conducted first by doing a BLAST (Basic Local Alignment Search Tool) search to compare and identify homologous sequences in the GenBank (22). We carried out comparative analyses of the aligned nucleotide sequences and predicted viral amino acids encoded by the nucleotides, using MEGA X. Whole-genome sequences of 101 WNV isolates were downloaded from GenBank (Supplementary Table 1). Using MAFFT (23) software, we aligned the reference sequences with seven sequences from our study, and we used Block Mapping and Gathering with Entropy (BMGE) (24) to trim the aligned sequences. We used PhyML 3.0 (25) to construct a phylogenetic maximum-likelihood tree with the default parameters, and the tree was displayed and annotated in Interactive Tree of Life (iTol) v5 (26).

## 3. Results

### 3.1. West Nile virus seroprevalence

**Figure 2:**
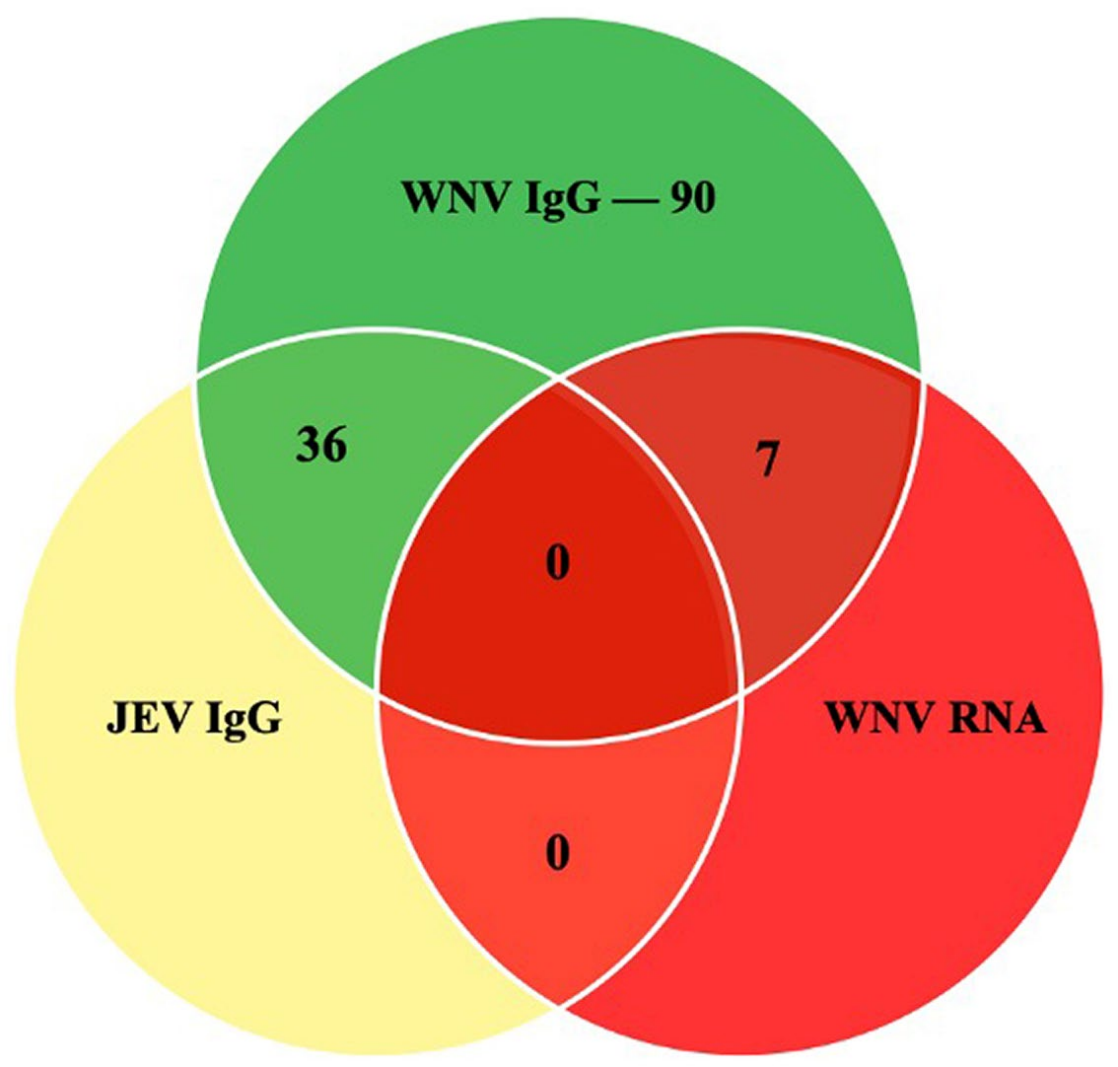
The Venn diagram shows the number of samples that were positive in the *WNV C-ELISA, JEV DAV-ELISA and the RT-PCR assays*.

In this study, 203 serum samples from three species were analyzed, out of which 90 were seropositive for WNV IgG (Figure 2). Table 1 shows the overall distribution of samples tested from all species and the number of positive samples from each location per species.

**Table 1:** WNV antibody and RNA detection according to species and sampling location Species.

Species	Location	No. of Samples	Positive WNV C-ELISA	Percentage seropositive	RT-PCR positive	Percentage RT-PCR positive
Cattle	Lenggeng	7	2	28.6	0	0
	Pulau Meranti	3	3	100	0	0
	Semenyih	19	8	42	0	0
	Serdang	54	14	25.9	0	0
Goat	Hulu Langat	29	14	48.3	0	0
Horse	Putrajaya	58	28	48.3	3	5.2
	Bukit Kiara	11	8	72.7	0	0
	Cheras	13	10	76.9	4	23.1
	Serdang	9	3	33.3	0	0
Total		203	90		7	

WNV antibodies were detected from all species and locations in this study. The seroprevalence in cattle was 32.5% (27/83) at 95% CI (0.2342–0.4319), in goats 48.3% (14/29) at 95% CI (0.3139–0.6557) and in the horses, the seroprevalence was 53.9% (49/91) at 95% CI (0.4366–0.6373). All the cattle samples from Pulau Meranti (3/3) were WNV seropositive, 25.9% (14/54) were positive in Serdang, 28.6% (2/7) were positive in Lenggeng, and 42.1% (8/19) were positive in Semenyih. All goat samples were from Hulu Langat. The recorded seroprevalence in horses was 76.9% (10/13) in Cheras, 72.2% (8/11) in Bukit Kiara, 48.2% (28/58) in Putrajaya and 33.3 (3/9) in Serdang.

### 3.2. Japanese encephalitis virus seroprevalence

In total, 36 samples from this study were positive for the cross-reactive Japanese encephalitis virus antibodies, representing 17.7% of the tested samples. The seropositivity in the horse was the highest, with JEV antibodies detectable in 34 animals (37%). In the cattle, two samples were positive (2.4%), and none of the goats had detectable JEV antibodies.

### 3.3. Molecular prevalence of WNV

**Figure 3:**
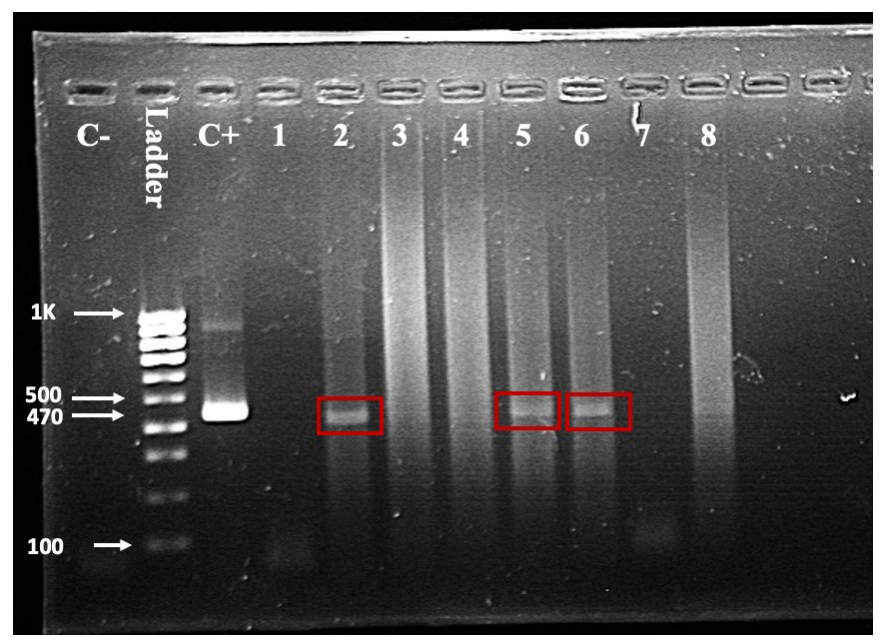
One-step RT-PCR with RNA from horse nasopharyngeal swab. A band in the positive control (C+) lane at 470 bp and the absence of a band in the negative control (C-) lane validates the reaction. Test samples are in the lanes labelled one through eight. Bands indicating a positive test are visible in lanes 2, 5, and 6.

Seven of the 203 (3.5%) samples analyzed using RT-PCR were positive (Figure 3). No positive RT-PCR test was recorded in the cattle and goats. Table 1 shows the distribution of the RT-PCR test based on species and location of sampling. The molecular prevalence of WNV in the horse was 7.69% at 95% CI (0.077–0.1503). The positive samples were from Cheras (*n* = 4) and Putrajaya (*n* = 3). Two mares were RT-PCR positive among the horses, one each from Cheras and Putrajaya. Three geldings from Cheras and two from Putrajaya were positive. The PCR-positive animals were also positive for the WNV C-ELISA test, and all the animals were healthy.

### 3.4. Partial sequencing and phylogenetic analysis

**Figure 4:**
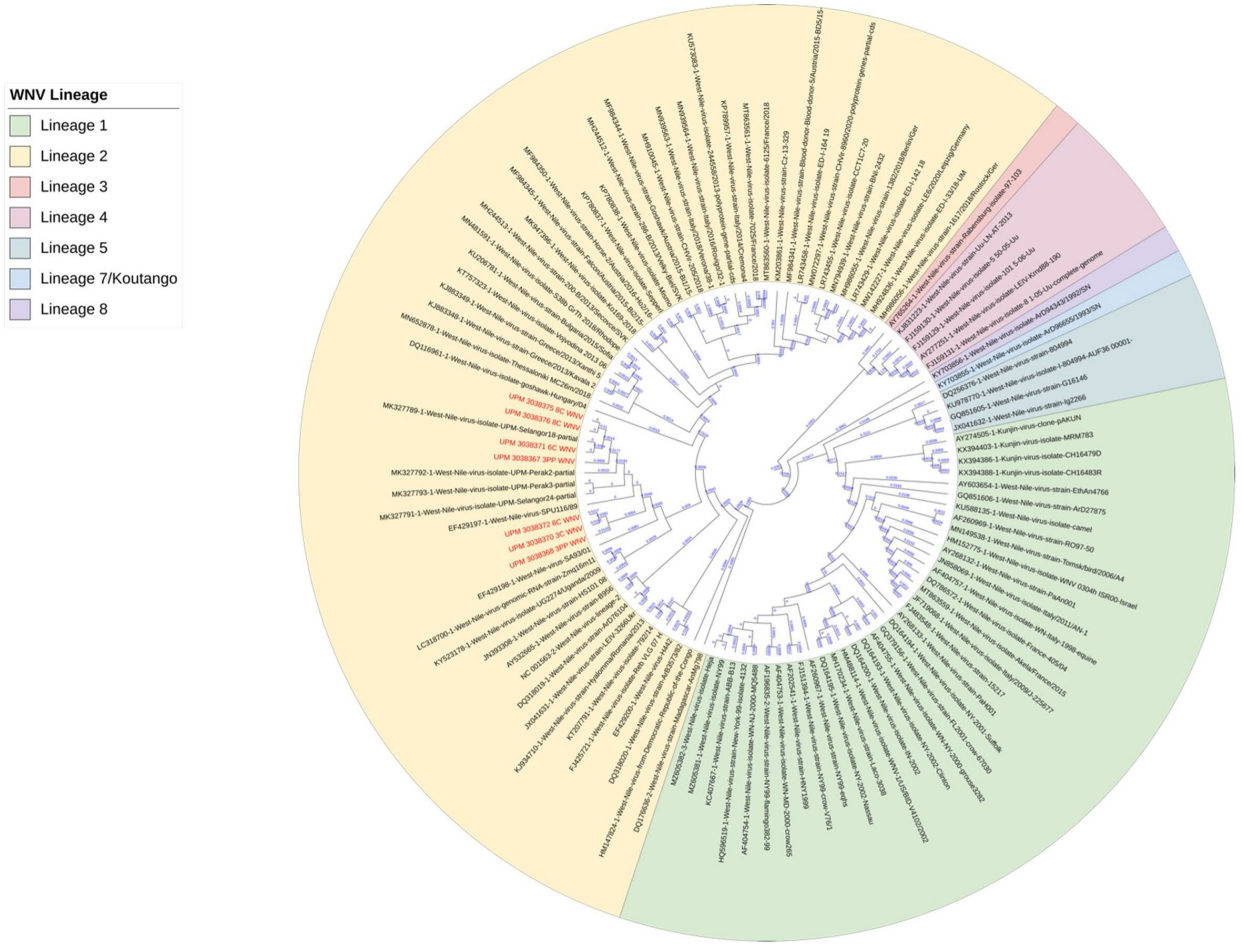
Maximum-likelihood phylogenetic tree of WNV isolates based on the conserved segment between the capsid (C) and pre-membranes (prM) genes in the WNV genome. 101 WNV sequences were compared to the seven sequences from this study. Different lineages of WNV are color-coded. All seven sequences from this study (in red text) clustered in lineage 2 (yellow highlight).

**Figure 5:**
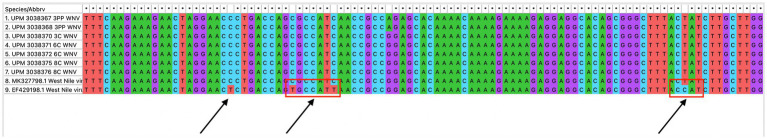
Multiple sequence alignment of the conserved region of WNV isolates from this study (seven isolates), a closely related isolate from Malaysia wild birds and a neurovirulent strain from South Africa. Variations in the nucleotide sequence are indicated with a red box.

**Figure 6:**
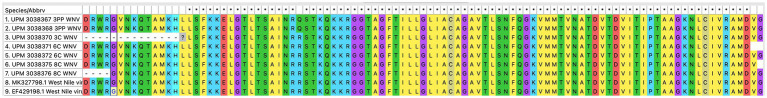
Translated amino acid sequences from the multiple sequence alignment of the conserved region of WNV isolates from this study (seven isolates), a closely related isolate from Malaysia wild birds and a neurovirulent strain from South Africa.

The seven sequences from our study all clustered into the lineage 2 clade of WNV based on the maximum-likelihood phylogenetic analysis of the conserved region in the WNV genome (Figure 4). Our WNV isolates are closely related to neuroinvasive strains of WNV from Africa, as well as WNV isolates from Malaysian wild birds. The nucleotide sequence comparison between the isolates from this study and two closely related lineage 2 WNV isolates; a human neuroinvasive strain from South Africa (GenBank accession number EF429198) and a Malaysian strain from wild birds (GenBank accession number MK327798.1) showed little variation with the South Africa strain (Figure 5). However, these substituted nucleotides appeared to have no effect on the encoded amino acids (Figure 6).

## 4. Discussion

West Nile virus is the most widely disseminated cause of arboviral neurological disease globally (3). It spreads readily in the presence of competent vectors, as well as reservoir and amplifying hosts. The virus naturally circulates between ornithophilic mosquitos, particularly those of the Culex genus, and avian species, although other vertebrate species, including mammals and reptiles, can become infected (27). WNV vectors are abundant in Malaysia contributing to possible risk of WNV transmission between reservoir and susceptible animals and humans (28–30). The tropical climatic condition in Malaysia provides all-year breeding opportunities for mosquitos. Their proximity to humans and livestock ensures an alternate source of nourishment, which could also lead to infectious agents’ spill-over into livestock populations. In addition to the avian species indigenous to Malaysia, several wild birds’ migratory routes pass through the country, providing an avenue for contact between these species and with Culex mosquitos that could lead to transmission of infectious agents. West Nile virus infection in domesticated mammals has not been investigated in Malaysia despite evidence of the circulation of the virus in the country among mosquitos, captive birds, humans, wild birds, bats, macaques and pigs (19–21, 31–33). Although no clinical infection has so far been reported in either humans or animals, the continued detection of WNV antibodies and viral RNA in these species is an indication that the virus is likely circulating in Malaysia.

This study was carried out on farms and stables in animals vulnerable to mosquito bites. The ruminants were managed semi-intensively, with the housing designed primarily to protect the animals against unfavorable weather conditions. The horses were managed intensively in stables that had no protection against mosquitos. All the animals that were sampled for this study were apparently in good health.

Viral infections in immuno-competent animals elicit an immune response characterized by the production of virus-specific antibodies (34). The detection of these antibodies can be an indication of an active or a past infection with the virus. The commercial competitive ELISA kit used in this study detects WNV IgG across different species. In this study, 90 out of the 203 animals had detectable WNV IgG in their serum. We detected WNV antibodies in the serum of cattle, goats and horses. The WNV seroprevalence varied among the different species, with cattle having a prevalence of 32.5% (27/83), goats with 48.3% (14/29) and the horse 53.9% (49/91).

WNV infection is not common in ruminants. Few studies have reported WNV infection in ruminants, with clinical illness recorded in few animals and in animals experimentally infected with virulent strains of WNV (16, 17, 35). Selim and Abdelhady, in a study involving ruminants in Egypt, reported similar varied WNV seroprevalences among the different species (12). However, the prevalence in that study was considerably lower than those reported in our study. Another study in Nigeria also reported the detection of WNV antibodies in ruminants, including camels, goats, cattle and sheep (36). Like our study, the author reported varied WNV seroprevalence among the different species of ruminants, although the antiquated WNV hemagglutination-inhibition (HI) antibody test was used in that study.

Among mammals, the horse seems to be the most severely infected species with WNV, with up to 20% of infected horses developing clinical symptoms, which often progresses to fatal neurological disease (13). Compared with the literature, the prevalence recorded in the horse in this study is unremarkable. Seroprevalence higher than 80% has been reported in apparently healthy horses in Nigeria (37). Schvartz et al. (38), in their study, reported a WNV prevalence of 84.1% in Israel and reported that horses kept in stalls and stables were more likely to be WNV seroprevalence than those kept on pasture. The serological result from this study reveals prior contact between the domesticated animals we tested and WNV. Because anti-WNV IgG has low avidity during the acute phase of the infection and binds more specifically with the maturation of more robust B-cell IgG clones (39, 40), these results could mean that the sampled animals were previously exposed to the virus and had recovered.

Accurate serological diagnosis of WNV can be challenging due to the broad serological cross-reactivity with other closely related flaviviruses. We found 36 of our WNV-positive samples, or 17.7%, to also be positive for the cross-reactive Japanese encephalitis virus. This is lower than the prevalence reported among livestock in high-risk areas in Malaysia where the reported JEV prevalence in cattle was 32%, and even higher prevalence at 80 and 44% in dogs and pigs, respectively, (41). The virus neutralization test (VNT) can distinguish between closely related flaviviruses and give a more precise prevalence of WNV. Unfortunately, due to the cost, time, and containment requirements of the VNT, we could not use the assay in our study.

Additionally, we detected WNV RNA in the nasopharyngeal swab from some sampled animals. In this study, the molecular prevalence of WNV was 3.44% (7/203) among all three species. In the horse, the only species in this study with a positive RT-PCR result, the molecular prevalence was 7.69% (7/91). WNV infection in cattle and goats is not common, and in this study, none of these two species of ruminants was RT-PCR positive for WNV. In a different study spanning 7 years and 1,407 animals that had developed neurological illness in South Africa, one cow and one goat were positive for the WNV RT-PCR test (42). The findings are also different from those reported by Ain-Najwa et al., where the seroprevalence of WNV in Malaysian wildlife and birds were 29.6 and 18.7%, respectively (19, 20). Those studies also reported a higher molecular prevalence of 12.2% in bats and 15% in birds. Our RT-PCR test results confirm recent WNV infection in the tested animals. Unlike the antibodies that can persist and remain detectable long after exposure and recovery from WNV infection, the virus is only detectable during the transient viremic phase of the infection or shortly afterwards (43).

Phylogenetic analysis of the sequences from our study showed a close relationship with previously sequenced isolates from Malaysian wild birds. Additionally, the sequences were closely grouped with two virulent WNV strains that were isolated from human patients, one of whom had died from necrotic hepatitis (isolate with accession number EF429197), and a second patient who was ill and had developed fever, rash, myalgia, and encephalitis (isolate with accession number EF429198) (44). However, none of the animals in our study, including those positive in the RT-PCR test, had any signs of disease. This finding suggests that the strains of WNV circulating in Malaysia, despite being phylogenetically related to virulent strains of the virus, are attenuated. The analysis of the nucleotide sequence as well as the translated amino acid sequence of the WNV isolates revealed very little difference between the isolates from this study and other closely related WNV isolates.

Our study adds to the evidence of WNV circulation in Malaysia among animals. WNV IgG antibodies were demonstrated in all the species studied, revealing prior exposure to the animals to the virus. Additionally, WNV RNA was detected in some of the horses, indicating a recent or ongoing infection in those animals. All the animals in this study were notably healthy, despite the phylogenetic similarity between the isolates and a virulent strain of WNV previously isolated from a human patient. The abundance of the WNV vector in Malaysia provides ample opportunity for WNV transmission to livestock in the vicinity of the vectors since these mosquito species readily feed on other vertebrate species in addition to their natural food source, which are the reservoirs of WNV.

## 5. Conclusion

This is the first report of WNV detection in domesticated mammals in Malaysia. Our study found a WNV seropositivity in cattle, goats and horses in Malaysia, likely due to the availability of these animals to be fed on by the WNV vector. These findings add to current reports of WNV circulation in wildlife, birds and other livestock in Malaysia and highlight the need for increased vigilance to prevent an outbreak of WN disease in Malaysia. Further studies utilizing neutralization assays and samples other than nasopharyngeal swabs will provide more in-depth information of WNV in these species in Malaysia.

## Data availability statement

The original contributions presented in the study are included in the article/supplementary material, further inquiries can be directed to the corresponding author.

## Ethics statement

The study was reviewed and approved by the Institutional Animal Care and Use Committee (IACUC) of the Universiti Putra Malaysia (IACUC approval number: UPM/IACUC/AUP/-R043/2017). Written informed consent was obtained from the owners of the animals sampled in this study.

## Author contributions

MM: writing—original draft preparation, investigation, methodology, and software. AY: supervision, writing—original draft preparation, methodology, conceptualization, and writing—reviewing and editing. SR, MN, OT, N-FH, SA, and HM: conceptualization and supervision. MA-N and JN: software and editing. All authors have read and agreed to the published version of the manuscript.

## Funding

This work is funded by Universiti Putra Malaysia Grantmanship - Geran Putra Berimpak (UPM.RMC.800/2/2/4-GPB-9702300) (ARY) and the Ministry of Higher Education Grant, Malaysia – France Bilateral Research Collaboration 2021 (MATCH 2021) with the grant number KPT MATCH/2021/5540495 (ARY) under the project In Vitro Differential Neuropathogenicity of Nonstructural Proteins (NSPs) of West Nile Virus isolated from Southeast Asia and Europe. The funders had no involvement in the study’s design, data collection and analysis, publication decision, or manuscript preparation.

## Conflict of interest

The authors declare that the research was conducted in the absence of any commercial or financial relationships that could be construed as a potential conflict of interest.

## Publisher’s note

All claims expressed in this article are solely those of the authors and do not necessarily represent those of their affiliated organizations, or those of the publisher, the editors and the reviewers. Any product that may be evaluated in this article, or claim that may be made by its manufacturer, is not guaranteed or endorsed by the publisher.
