# Supplementary material for: Serological and molecular surveillance of West Nile virus in domesticated mammals of peninsular Malaysia
Source: Front Vet Sci. 2023 Jun 29;10:1126199. doi: 10.3389/fvets.2023.1126199 (PMC10343450; doi:10.3389/fvets.2023.1126199)
Supplement: Supplementary file 1 [file Table_1.pdf]

Supplementary Table 1 List of WNV sequences from the GenBank used in the phylogenetic analysis

No.	GenBank Accession number	Country of Collection	Year of Collection	Lineage	Host
1	KJ831223	Austria	2013	4	*Uranotaenia unguiculata*
2	FJ159131	Russia	2005	4	*Uranotaenia unguiculata*
3	AY277251	Russia	1998	4	*Dermacentor marginatus*
4	FJ159130	Russia	2005	4	*Uranotaenia unguiculata*
5	FJ159129	Russia	2005	4	*Uranotaenia unguiculata*
6	AY765264	Czech Republic	2005	3	*Culex pipiens*
7	KY703856	Senegal	1992	8	*Culex perfuscus*
8	GQ851605	India	1957	5	Mosquito
9	DQ256376	India	1980	5	*Homo sapiens*
10	JX041632	India	1955	5	*Culex vishnui*
11	KU978770	India	1988	5	*Homo sapiens*
12	KX394403	Australia	1963	1/KUN*	*Anopheles bancroftii*
13	AY274505	Australia	2003	1/KUN*	-
14	KX394388	Australia	1974	1/KUN*	*Culex annulirostris*
15	KX394386	Australia	1974	1/KUN*	*Culex annulirostris*
16	AY603654	Ethiopia	2004	1	-
17	GQ851606	Senegal	1979	1	-
18	JN858069	Italy	2011	1	*Homo sapiens*
19	KU588135	UAE	2015	1	*Camelus dromedarius*
20	AF260969	Romania	1996	1	*Culex pipiens*
21	MN149538	Russia	2006	1	*Acrocephalus dumetorum*
22	AF404757	Italy	1998	1	Equine
23	AY268132	France	2000	1	Horse
24	HM152775	Israel	2000	1	*Homo sapiens*
25	MT863559	France	2015	1	*Equus caballus*
26	DQ786572	France	2004	1	*Passer domesticus*
27	FJ483548	Italy	2008	1	Magpie
28	JF719068	Italy	2009	1	Jay
29	AY268133	Tunisia	1997	1	*Homo sapiens*

30	GQ379156	USA	2001	1	Crow
31	DQ164194	USA	2001	1	American crow
32	HM488114	USA	2002	1	*Aedes cinereus*
33	DQ164193	USA	2002	1	American crow
34	DQ164200	USA	2002	1	*Homo sapiens*
35	DQ164195	USA	2002	1	*Culex pipiens/restuans*
36	MH170234	USA	2003	1	*Pica hudsonia*
37	AF404755	USA	2000	1	Ruffed grouse
38	AF260967	USA	1999	1	Horse
39	FJ151394	USA	1999	1	Crow
40	AF196835	USA	1999	1	Flamingo
41	AF404753	USA	2000	1	Crow
42	KC407667	Spain	2007	1	Mouse
43	MZ605382	Hungary	2004	1	Goshawk
44	MZ605381	USA	1999	1	Flamingo
45	AF202541	USA	1999	1	*Homo sapiens*
46	AF404754	USA	2000	1	*Culex pipiens*
47	HQ596519	USA	1999	1	Crow
48	MH924836	Germany	2018	2	*Strix nebulosa*
49	MH986056	Germany	2018	2	*Turdus merula*
50	LR743429	Germany	2018	2	Tawny Owl
51	MW142227	Germany	2020	2	*Homo sapiens*
52	MH986055	Germany	2018	2	*Turdus merula*
53	MN794939	Germany	2019	2	*Prunella modularis*
54	LR743455	Germany	2019	2	*Culex pipiens*
55	LR743458	Germany	2019	2	*Bubo scandiacus*
56	MW072297	Germany	2020	2	*Homo sapiens*
57	MF984341	Austria	2015	2	*Homo sapiens*
58	KM203861	Czech Republic	2013	2	*Culex modestus*
59	MT863560	France	2018	2	*Buteo buteo*
60	MT863561	France	2018	2	*Accipiter gentilis*
61	KP789957	Italy	2014	2	*Homo sapiens*
62	KU573083	Italy	2013	2	*Culex pipiens*

63	MN939563	Italy	2018	2	*Homo sapiens*
64	MN939564	Italy	2016	2	*Homo sapiens*
65	MH910045	Germany	2018	2	*Homo sapiens*
66	MF984344	Austria	2015	2	Goshawk
67	MH244512	Slovakia	2013	2	Northern goshawk
68	KP780837	Austria	2008	2	*Nestor notabilis* (Kea)
69	KP780838	Austria	2009	2	*Nestor notabilis* (Kea)
70	MF984345	Austria	2015	2	Falcon
71	MF984350	Austria	2016	2	Horse
72	MK947396	Slovenia	2018	2	Mosquito
73	MH244513	Slovakia	2013	2	Eurasian sparrow hawk
74	KU206781	Bulgaria	2015	2	*Homo sapiens*
74	MN481591	Greece	2018	2	Dog
76	KT757323	Serbia	2013	2	*Culex pipiens*
77	KJ883348	Greece	2013	2	*Homo sapiens*
78	KJ883349	Greece	2013	2	*Homo sapiens*
79	MN652878	Greece	2018	2	*Culex spp.*
80	DQ116961	Hungary	2004	2	Goshawk
81	MK327789	Malaysia	2017	2	Wild bird
82	MK327792	Malaysia	2017	2	Wild bird
83	MK327791	Malaysia	2017	2	Wild bird
84	MK327793	Malaysia	2017	2	Wild bird
85	EF429197	South Africa	1989	2	*Homo sapiens*
86	KY523178	Uganda	2009	2	*Culex neavei*
87	LC318700	Zambia	2016	2	*Culex quinquefasciatus*
88	JN393308	South Africa	2008	2	Horse
89	EF429198	South Africa	2001	2	*Homo sapiens*
90	JX041631	Ukraine	1980	2	Bird
91	DQ318019	Senegal	2006	2	-
92	NC_001563	USA	2019	2	Clone*
93	AY532665	USA	2004	2	Clone**
94	KT207791	Italy	2014	2	Mosquito
95	KJ934710	Romania	2013	2	*Hyalomma marginatum*

96	FJ425721	Russia	2007	2	*Homo sapiens*
97	DQ318020	CAR	2005	2	-
98	EF429200	South Africa	1958	2	*Homo sapiens*
99	HM147824	DRC	1958	2	-
100	DQ176636	Madagascar	1978	2	*Coracopsis vasa*
101	KY703855	Senegal	1993	7/Koutango	*Rhipicephalus guilhoni*

Note: KUN* – Kunjin; Clone* – NCBI Reference Sequence West Nile virus lineage 2; Clone** – Suckling mouse brain passage 2; CAR – Central African Republic; DRC – Democratic Republic of Congo; UAE – United Arab Emirates; USA – United States of America.
